# Description of the Characteristics of Five Bedding Materials and Association With Bulk Tank Milk Quality on Five New York Dairy Herds

**DOI:** 10.3389/fvets.2021.636833

**Published:** 2021-04-30

**Authors:** Valeria M. Alanis, Michael Zurakowski, Deb Pawloski, Tiago Tomazi, Daryl V. Nydam, Paula A. Ospina

**Affiliations:** ^1^Department of Population Medicine and Diagnostic Sciences, College of Veterinary Medicine, Cornell University, Ithaca, NY, United States; ^2^Department of Population Medicine and Diagnostic Sciences, College of Veterinary Medicine, Cornell University, Cobleskill, NY, United States; ^3^Lechear Limited Liability Company, King Ferry, New York, NY, United States

**Keywords:** bacteria counts, milk quality, environmental mastitis, bulk tank milk, bedding material

## Abstract

Environmental mastitis represents a major challenge on dairy farms where contagious pathogens are controlled by improved milking procedures. Therefore, research focused on the environment is important to improve udder health programs. The objectives of this prospective and descriptive study were to (1) describe bedding bacterial counts, pH, and dry matter (DM) of five different bedding types (organic: manure solids, straw, paper fiber; inorganic: sand, recycled sand) and (2) explore the association between bedding bacterial counts with bulk tank milk quality. This study took place within five conveniently selected commercial dairy herds, each with a predominant bedding material in lactating pens. Bedding samples (used *n* = 237; fresh *n* = 53) were collected monthly from July 2018 to July 2019 following a standard operating procedure (SOP) to minimize sampling variability. Additionally, a bulk tank (BT) milk sample (*n* = 40) was collected on the same day unless milk had been picked up prior to arrival. Both BT and bedding samples were submitted to the laboratory for culture and bacterial identification and quantification of *Streptococcus* spp, coliforms, and non-coliforms as well as detection of several pathogens of mastitis importance. Somatic cell count was evaluated in BT samples. Within bedding type, the correlation between bedding characteristics and bacterial counts in bedding was evaluated using Pearson correlation. Within bedding type, the correlation between bacterial counts in bedding samples and bacterial counts in BT were determined. The Kruskal–Wallis test was used to evaluate the bacterial count by bedding type and to evaluate BT somatic cell count differences based on bedding type. In fresh bedding, bacterial counts were generally higher for manure solids for all bacterial groups compared with other materials. In used samples, organic materials had the highest levels of all bacterial groups. The proportion of samples with detectable organisms of mastitis importance varied within and among herds in both bedding and BT samples throughout the study period. In bedding samples, a higher DM content had the lowest levels of bacterial growth compared with those with lower DM content. Most bedding samples were on the alkaline side within a pH range of 8–11. No relationship between bacterial counts and pH was observed. No associations between BT bacteria counts and bedding bacterial counts were observed. No association between bulk tank somatic cell counts based on bedding type were observed. Despite using an SOP for bedding sampling in an effort to consistently collect samples, we still observed a large amount of variability both within and among bedding samples. This variability may have obscured any potential association between BT milk quality and bedding type.

## Introduction

As a multifactorial disease, bovine mastitis is one of the most complex, frequent, and costly diseases of dairy herds associated with decreased milk yield and quality (1–4). Research shows that coliform and *Streptococcus* spp pathogens cause impactful milk losses (3–5) and that these losses vary between primiparous and multiparous cows. Raw milk with high somatic cell count (SCC) often has higher lipolysis and proteolysis than in low SCC milk and also has effects in pasteurized milk, such as decreasing shelf life and sensory defects, including rancidity, bitterness, and astringency (6). In the last years, there have been some changes in the distribution and patterns of mastitis in dairy herds in developed countries with an important decrease of cows with contagious forms of mastitis but persistent environmental forms (7–10).

Coliforms (including *Escherichia* spp, *Klebsiella* spp, and other Gram-negative bacteria), Streptococcus species (including *Streptococcus uberis* and *Streptococcus dysgalactiae*), and non-aureus *Staphylococcus* are among the most common environmental bacteria causing mastitis in U.S. dairy herds (USDA, 2014). This distribution of mastitis pathogens was also identified in a recent study from eight commercial herds in New York (11). Additionally, cows with at least one clinical mastitis case due to environmental pathogens, such as *E. coli, Klebsiella* spp, and *T. pyogenes*, have greater risks of culling (12) compared with non-mastitic cows. Further, Gram-negative cases increased the risk of mortality as stated in a study from 30,233 lactations in cows of seven dairy farms in New York State (13).

These environmental mastitis pathogens have been isolated from bedding materials, soil, rumen, feces, vulva, lips, nares, and feed samples (14–17), which demonstrates their nearly ubiquitous risk to environmental and teat end contamination. Like any other types of bacteria, they require appropriate moisture, temperature, and nutrients to live. Appropriate conditions are often present on dairy farms to allow bacterial numbers to increase. Therefore, the number of these bacteria on teat skin is a reflection of the cow's exposure to the contaminating environment (18). Bedding material itself has physical and biochemical properties that support bacterial growth along with external factors that influence it (19).

Extensive research demonstrates that both heifers and cows need 12–14 h of lying daily and that they prioritize it over other activities (20, 21). Considering this strong behavioral need to rest, a fundamental issue to consider is bedding materials that provide adequate cushion and also that can reduce udder and teat exposure to environmental pathogens. Exposure to these pathogens when the cow lies down could result in intramammary infections with a possible mastitis outcome (18). Several studies show that bacteria can be transferred between the lying surface and the teats (22–25). Because environmental pathogens are highly influenced by management practices, such as the housing system, cow comfort, manure collection method, proper bedding, and pen cleanliness (26, 27), one of the most difficult dairy farm challenges is to minimize the level of exposure to environmental mastitis pathogens at the teat level between milkings to maintain good udder hygiene.

Few studies focus on the association between bedding material and bulk tank (BT) milk quality (i.e., bacterial load and somatic cell counts). Among these few studies, there have been few consistent results. One prospective study using data from BT test results from 325 dairy herds in Wisconsin using the same bedding in all pens during the two-year study period (28) shows that total bacterial counts in the BT were not associated with bedding type, but bulk milk somatic cell score (BTSLS) was lower for farms using inorganic materials.

A cross-sectional study using data from 125 herds in the United Kingdom (29) show no significant differences between bedding material in bacterial counts in milk for any of the organisms studied and no significant correlations between bacterial load in used bedding and milk. More recently, another cross-sectional study using data from 167 herds from 17 states in the United States (30) shows a wide variation of pathogen load in bedding among farms with organic material bedding showing the highest coliform levels compared with inorganic materials and manure solids showing the highest counts for streptococci-like organisms. They establish a guide for monitoring bedding hygiene in commonly used organic and inorganic bedding. Looking at another aspect of milk quality, research focused on food safety shows that bedding management practices (e.g., re-bedding frequency, raking frequency) were associated with mesophilic and thermophilic spore levels, and used organic bedding spore levels were positively related to those in BT milk (31).

The objectives of this prospective and descriptive study with repeated measures were to (1) describe the variability in bedding bacterial counts, pH, and dry matter (DM) of five different bedding types (manure solids, sand, straw, paper fiber, and recycled sand) and (2) explore the association between bedding bacterial counts with BT milk quality in five conveniently selected New York dairy farms using one of five bedding materials in lactating pens.

## Materials and Methods

### Herd Selection and Sample Collection

Five commercial dairy herds in central New York State with an average herd size of approximately 1,400 cows (ranging from 838 to 2,050) were conveniently selected based on the willingness of the producers to participate and the proximity of the herds to the Quality Milk Production Services laboratory (QMPS) at the Animal Health Diagnostic Center, Cornell University (Ithaca, New York). Each herd used a predominant bedding material for lactating pens: manure solids (MS), paper fiber (PF), straw (ST), recycled sand (RS), or sand (SD).

All herds used Dairy Comp 305 (DC305; Ag Valley Software) as the management software. Participating herds used a well-established milking routine, and every case of mastitis was identified by trained on-farm personnel, who collected all milk samples from all quarters with visibly abnormal milk, stored in a refrigerator (≅4°C), and saved information in DC305. These milk samples were submitted to the QMPS laboratory for culture and matrix-assisted laser desorption/ionization time-of-flight (MALDI-TOF) identification. These herds also had a regular DHIA testing program (monthly individual SCC and linear score) and were fed a balanced total mixed ration (TMR).

Farms were visited once monthly for a period of 6 to 12 months from July 2018 to July 2019 by the same observer. The sample collection period among the herds varied in one herd because they changed bedding type mid-study. At each visit, used and fresh bedding samples were collected as well as a BT sample and a DC305 backup.

### Herd Bedding Practices

The herd using RS used a modified plug-flow aerobic digestion system with recirculation and mixing and a multistage SD separation system. The herd using MS used a screw press as a manure separation system. Herds using PF, SD, and ST purchased the material and stored it in a clean and dry storage area inside the herd.

They were also asked to notify investigators of any changes to these management practices during the study.

### Bedding Samples

The samples were collected once a month from lactation pens. A standard operating procedure (SOP) was followed to minimize sampling variability. The day that the fresh bedding was due to be applied and after the routine cleaning, used bedding from three to five stalls from each pen was collected. Wearing clean disposable gloves, samples were collected from a 60 cm x 60 cm area, avoiding any manure spots, where the udder would touch the stall after scraping 3 cm off the top of the bedding material. Samples were transferred to a new one-quart storage freezer bag.

Using new and clean disposable gloves, fresh bedding samples were collected after asking the worker to dump extra bedding material in five stalls distributed throughout the pen. Fresh bedding was collected from the top of this pile to form a combined sample. The sample was transferred to a new one-quart storage freezer bag.

Each used and fresh sample bag was labeled with the herd name, pen number, and date. Samples were placed in ice coolers, transported the same day within 2 h after sampling, and frozen at−18°C for up to 4 weeks for analysis at QMPS.

### BT Milk Samples

Unless milk had been picked up prior to arrival, the same day bedding was sampled, one BT sample was collected directly from the BT using a clean and sanitized dipper into a 10-ml vial. Sampling was performed following the Dairy Practices Council (DPC) guidelines (i.e., mechanically agitate the milk for at least 5 min until sufficient homogeneity is obtained and 10 min for tanks larger than 1,500 gallons). Each vial was labeled with the herd name and date. Samples were placed in ice coolers at 1°C, transported the same day within 2 h after sampling, and frozen at−18°C for up to 4 weeks for analysis at QMPS.

### Laboratory Analysis and Bacteria Quantification

Frozen bedding and BT samples were submitted for bacterial identification and quantification for *Streptococcus* spp, coliforms, and non-coliforms at QMPS as well as detection of other pathogens associated with mastitis.

#### Bedding Samples

Frozen samples were allowed to thaw at refrigeration temperature (2°C−8°C) for one to 4 h, depending on the bedding material to be analyzed. The sample was placed into a large, clean, zip-type bag that allowed thorough mixing and breaking up of any clumps. For ST samples, pieces were cut into approximately 2.5 cm in length using sterile scissors. Using a weight-verified scale, bedding material was weighed 10 ± 1% (9.90–10.10) grams into a stomacher bag (MS, SD, and PF) or sterile vial (RS, ST) by taking small subsamples from at least three random locations within the mixed sample. Then, 90 ml of sterile PBS was added to the 10-g test sample and mixed for 2 min using a stomacher set at blending speed 2 (two strokes/second) or vortex for 40 s at setting 7 (1,800 rpm) for vials. Approximately 10 ml of this suspension was decanted into an empty sterile dilution tube. This was the 10^−1^ dilution. The 10^−2^ dilution was made by vortexing the 10^−1^ dilution for a minimum of 4 s and removing 1 ml using a micropipette and adding it to 9 ml of PBS. This dilution process continued until the 10^−5^ dilution.

#### BT Samples

Frozen samples were allowed to thaw at refrigeration temperature 2°C−8°C and mixed thoroughly by shaking. The 10^−1^ dilution was made by removing 1 ml and adding it to 9 ml of PBS and vortex for a minimum of 5 s after a vortex has been achieved. This dilution process continued until the 10^−2^ dilution.

#### Plate Inoculation and Incubation Parameters (Bedding and BT Samples)

For each bedding and BT sample, 50 μl of each dilution was inoculated on different selective media. Edwards media was inoculated to test for *Streptococcus* spp and “streptococci-like” organisms. MacConkey media was inoculated to test for coliforms and non-coliforms. Hayflick media was inoculated with 50 μl of used bedding material from dilutions 10^−2^, 10^−3^, and 10^−4^ to test for mycoplasma and placed in a CO_2_ incubator. For BT samples, trypticase soy agar with 5.0% blood and 0.1% esculin media was inoculated to test for total count of all organisms (TBC).

In addition to the organisms that were quantified, the following organisms of mastitis significance were identified and counted as detected or not detected: *Staphylococcus aureus, Streptococcus agalactiae, E. coli, Klebsiella* spp, *Serratia* spp, *Pasteurella* spp, *Pseudomonas* spp, *Prototheca* spp, *Trueperella pyogenes*, yeast, mold, other fungi, and other microorganisms (*Listeria* spp, *Nocardia* spp, and *Salmonella* spp). Experienced technicians in microbiology used visual cues and biochemical tests (NMC, 2017) along with colony morphology of the plate to identify these pathogens. The presence of even one colony would be considered as detected.

Plates were incubated at 35°C−38°C. After 18–24 h of incubation, plates were observed using standard microbiology procedures. At 18–24 h, the lactose-positive, Gram-negatives colonies were counted and *E. coli* and *Klebsiella* were observed and recorded. Plates were placed back in the incubator at 35°C−38°C for an additional 18–24 h.

#### Bacteria Counts Calculation

Plates were removed from the incubator, and the number of colony-forming units (CFU; CFU/g for bedding samples and CFU/ml for BT samples) counted by an experienced laboratory technician from the dilution plate (up to 10^−5^ for bedding samples and up to 10^−2^ for BT samples) that presented 25–250 colonies whenever possible. All counts and the dilution plate were recorded in an internal form. The formulas used are as follows:

**Bedding:**

((A(1000/B)*9)/C)/(D/E)

$$\begin{array}{l} \left(\frac{\left(\frac{A\;CFU}{50\;\mu L}\right)\;*\left(\frac{1000\;L}{1\;mL}\right)}{\left(\frac{10\;g}{90\;mL}\right)}\;\right)\;*C\;*\left(\frac{E}{D}\right)\\ =\left(\left(\left(\left(\frac{A\;CFU}{50\;\mu L})\;*(\frac{1000\;L}{1\;mL}\right)\right)*(\frac{90\;mL}{10\;g})\right)*\left(10^{n}\right)\right)*\left(\frac{E}{D}\right)\\ =X\;\frac{CFU}{g} \end{array}$$

Where:

A = number of colonies (CFU)

B = inoculation volume = 50 μl

C = dilution factor, *n* (10^−*n*^)

D = dry weight (g)

E = wet weight (g)

**% moisture**

((A+B)-C)*100/10

Where:

A = empty dish

B = bedding weight (added to the dish to go into the oven)

C = after drying (dish + bedding)

**BT:**

***A(1000/B)/C***

Where:

A = number of colonies (CFU)

B = inoculation volume (μl)

C = dilution value of the plate counted or dilution factor, *n* (10^−*n*^).

#### Moisture Content (DM Content) Estimation

The drying dish was weighed. The scale was tared and 10 ± 1% (9.90–10.10) g of bedding material was added and evenly spread. The dish containing the 10 g of bedding was placed into the oven and dried for at least 4 h at 100 ± 10°C. After drying, the sample was weighed, and the total weight to two decimals was recorded.

#### pH Estimation

A flip-top vial was placed on the scale and tared and 10.00 g of bedding material was added by taking small subsamples from at least three random locations within the mixed sample. Next, 90 ml of deionized water was added using a 100-ml graduated cylinder and mixed well. The pH probe from a pH meter was verified with appropriate buffers (7 and 10 buffers for calibration as most bedding material fit that range). If a bedding material ended up with a lower pH after calibration with the 7 and 10 buffer, the pH meter was recalibrated using a 4 and 7 buffer. This probe was placed into the mixture, and pH was recorded to two decimals.

#### Somatic Cell Count

BT milk SCCs (BTSCC) were analyzed using a DeLaval cell counter (DCC). The DCC analyses were performed using samples at 10°C−40°C following the manufacturer's instructions. To transform BTSCC into BTSLS the following equation was applied: BTSLS = log2 (BTSCC/100) + 3.

### Statistical Analysis

Data collected and laboratory results were transferred to Excel spreadsheets (Microsoft Corp; Redmond, WA). Data were imported into R version 4.0.3 (RStudio: Integrated Development for R. RStudio, Inc., Boston, MA) to perform statistical analysis and to create the appropriate plots. All graphical representations were made using the ggplot2 package. The normality of continuous variables (i.e., bacteria counts) was visually assessed with density plots and quantile-quantile plots. These were not normally distributed; therefore, bacteria count values greater than zero were log10 transformed. When no bacteria were identified, a value of log10+1 CFU/g for bedding and log10+1 CFU/ml for BT was used, assuming that at least 10 CFU were present in a given sample. The decision to use this arbitrary value was due to the potential losses on each dilution before having the final count. An additional outcome was created in which the counts of each bacterial group isolated (*Streptococcus* spp, coliforms, and non-coliforms) in bedding samples were summed. This new outcome was named sum bacterial count (SBC).

Within bedding type, the correlation between bedding characteristics and bacterial counts in bedding were evaluated using Pearson correlation. For bacterial count analysis in bedding samples, the Kruskal–Wallis test was used to evaluate the differences based on bedding type running the kruskal.test function. When appropriate (meaning following a Kruskal–Wallis test at *P* < 0.05), Dunn's multiple comparison test among the five bedding materials and Bonferroni correction were used as a *post hoc* nonparametric test running the dunn.test function. Correlations between bedding characteristics (pH and DM) and bacterial counts were determined using the Pearson correlation coefficient running the cor.test function. Within bedding type, the correlation between bacterial counts in bedding samples and bacterial counts in BT were determined. The Kruskal–Wallis test was used to evaluate the bacterial count by bedding type and to evaluate BT somatic cell count differences based on bedding type.

For bacterial count analysis in BT samples and to evaluate differences between BTSLS based on bedding type; the Kruskal–Wallis test was used to evaluate the differences based on bedding type running the kruskal.test function. When appropriate (meaning following a Kruskal–Wallis test at – < 0.05), Dunn's multiple comparison test among the five bedding material and Bonferroni correction were used as a *post hoc* nonparametric test running the dunn.test function. The proportion of bedding and BT samples with detectable organisms from the list of pathogens of mastitis importance was also described. Correlation between bedding bacterial counts and bacterial counts was determined using the Pearson correlation coefficient running the cor.test function with the average value of used bedding samples per time point.

## Results

### Study Herds

The mean number of lactating cows was 1,400, and the daily mean milk production was 37 kg. The mean BTSCC was 130,000 cells/ml. All farms used a consistent milking routine with pre-dipping, foremilk stripping, and wiping teats with either cloth (MS, PF, RS, and SD) or paper towels (ST). All farms used iodine-based disinfectant solutions for pre-dipping and post-dipping. Basic farm descriptors, design, and management of bed descriptors are displayed in Table 1. Additionally, the results of cow positioning, bedding quantity, and quality can also be found in Table 1. Generally, most cows had adequate positioning (>70% except the MS herd with 25%).

**Table 1:** Herd characteristics from five conveniently selected New York dairy herds using one of five bedding materials in lactating pens.

**Herd**	**Bedding type**	**Milking cows (n)^a^**	**DIM^b^**	**Average milk (kg)^c^**	**Average SCC^d^**	**Type of stalls**	**Re-bedding frequency^e^**	**Rake/groom bedding surface frequency**	**Milking frequency per day**	**Milking parlor type/Number of stalls**	**Towel material**	**Dry-off routine**
A	Manure solids	2,050	189	41.7	143	Deep beds	Daily	3x daily	3x	Rotatory/80	Cloth	Blanket
B	Paper fiber	1,214	184	39.4	166	Mattress	Twice a week	2x daily	2x	Parallel/Double 18	Cloth	Blanket
C	Straw	838	161	32.2	211	Concrete	Daily	2x daily	2x	Parallel/Double 12	Paper	Blanket
D	Recycled sand	1,750	172	40.3	187	Deep beds	Weekly	3x daily	3x	Parallel/Double 17	Cloth	Selective
E	Sand	1,170	169	39.9	148	Deep beds	Weekly	3x daily	3x	Parallel/Double 18	Cloth	Blanket

a *Lactating cows monthly average*.

b *Average days in milk*.

c *Average of total daily milk produced (kg)*.

d *Test day average somatic cell count (SCC; × 1,000 cells/mL) over the year of study*.

e *Frequency of adding new bedding material to resting area and stalls*.

### Bacterial Counts in Bedding Samples

All collected samples were evaluated in the laboratory. Although the goal was to collect 12 fresh samples (one representative stall per month from each herd bedding type) and 60 used samples (five representative stalls per month from each herd bedding type) from 12 monthly visits (*n* = 360 total samples), only a total of 290 bedding samples (used *n* = 237; fresh *n* = 53) were collected for final analysis. The difference in the number of samples was due to lack of bedding available to sample (18 visits among herds) or equipment malfunction (12 visits among herds) on the follow-up visit. Due to cold storage space and laboratory time limitations, the number of used bedding samples collected from each farm visit was changed to three in the second half of the study. Last, we started sampling in the ST herd later compared with the other herds, which affected the final number of samples, and this herd changed bedding midway through the study, which severely limited the number of used samples of this bedding type. Thus, inferences from ST should be interpreted in light of the small number of observations. Comparatively the fresh samples were not as strongly impacted. The final analysis consisted of MS = 54 (used *n* = 44; fresh *n* = 10), PF = 86 (used *n* = 70; fresh *n* = 16), ST = 24 (used *n* = 18; fresh *n* = 6), RS = 74 (used *n* = 60; fresh *n* = 14), and SD = 52 (used *n* = 45; fresh *n* = 7).

**Figure 1:**
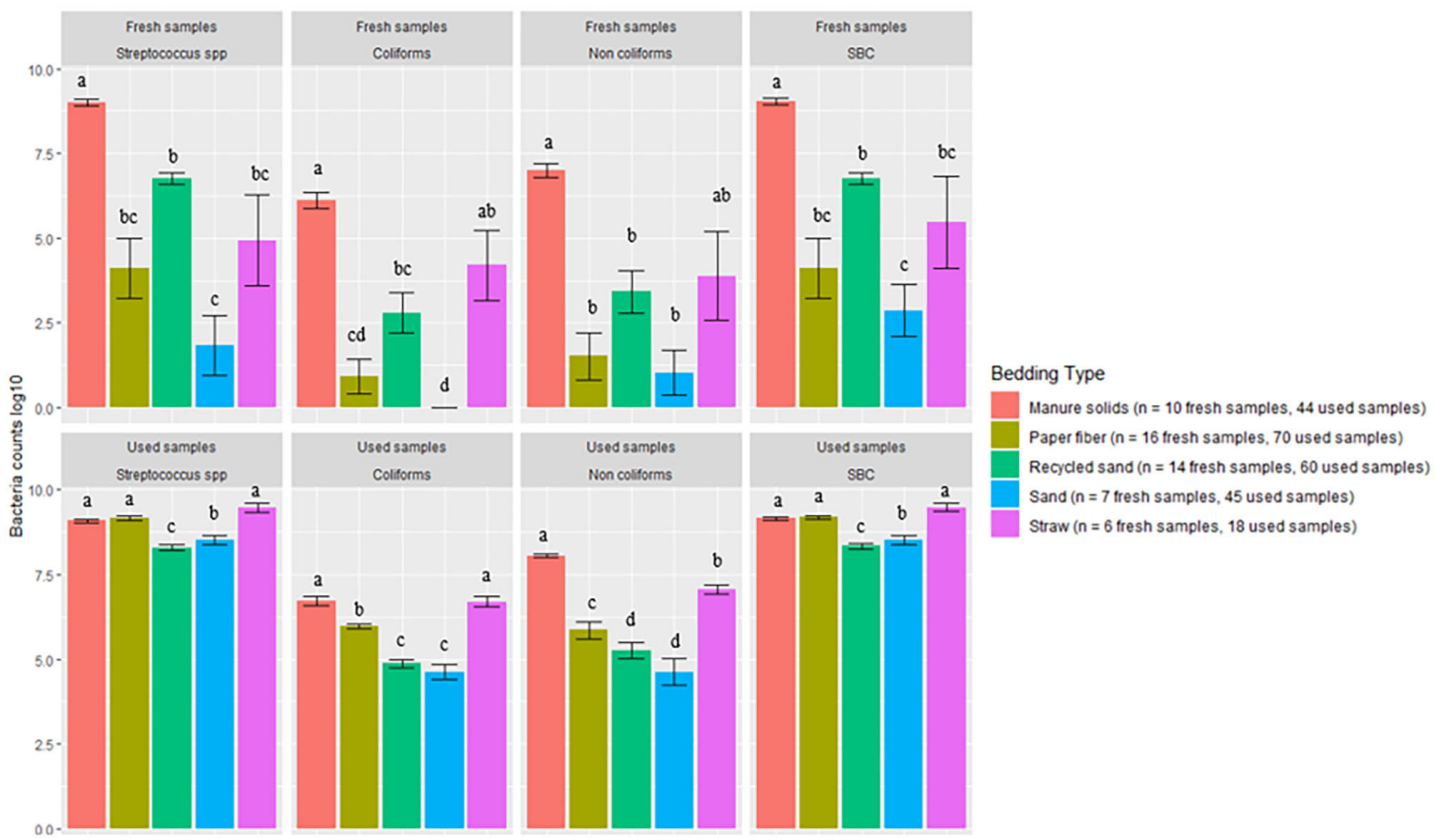
Average bacterial counts (log10 CFU/g) for fresh and used bedding samples collected from July 2018 to July 2019 from five conveniently selected New York dairy herds using one of five bedding materials in lactating pens. One fresh bedding sample and three to five used bedding samples were collected monthly following an SOP at each visit (unless there was no bedding available due to lack of supply or equipment malfunction on the follow-up visit). Error bars represent SD. The same letters are not different at *P* ≤ 0.01 (*P*-values adjusted for multiple contrasts). SBC (sum bacterial count) = Streptococcus spp, coliforms and non-coliforms summed.

Bacterial counts (log_10_ CFU/g) from fresh and used samples during the entire study period are summarized in Figure 1. The ST samples showed a wider variation on all bacterial counts compared with the other bedding types. The SD bedding type had four fresh samples with no detectable levels of *Streptococcus* spp and no detectable levels of coliforms.

There was a clear increase in bacterial counts in used bedding samples compared with fresh samples for all bedding materials. *Streptococcus* spp, coliform, and non-coliform counts in inorganic materials (RS and SD) were generally lower than in organic materials (MS, PF, and ST). For example, coliform counts were different between all bedding types, being the highest on ST, then equally highest on MS and ST, and equally lowest on RS and SD (MS vs. SD *P* < 0.0001, MS vs. RS *P* < 0.0001, ST vs. SD *P* < 0.0001, ST vs. RS *P* < 0.0001). All pairwise comparisons are shown in Figure 1. A similar relationship was seen with SBC counts in which inorganic materials were approximately 1 log_10_ less than the organic materials.

**Figure 2:**
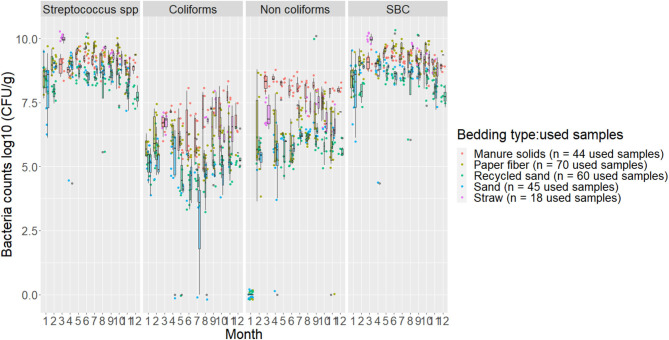
Boxplots showing 25th, 50th (median), and 75th percentiles of the distribution of bacteria counts (log10 CFU/g) for used bedding samples collected from July 2018 to July 2019 from five conveniently selected New York dairy herds using one of five bedding materials in lactating pens. Used bedding samples (three to five) were collected monthly following an SOP at each visit (unless there was no bedding available due to lack of supply or equipment malfunction on the follow-up visit).

The variability between used samples collected on the same day is illustrated in Figure 2.

### Detection of Specific Bacteria in Bedding

**Figure 3:**
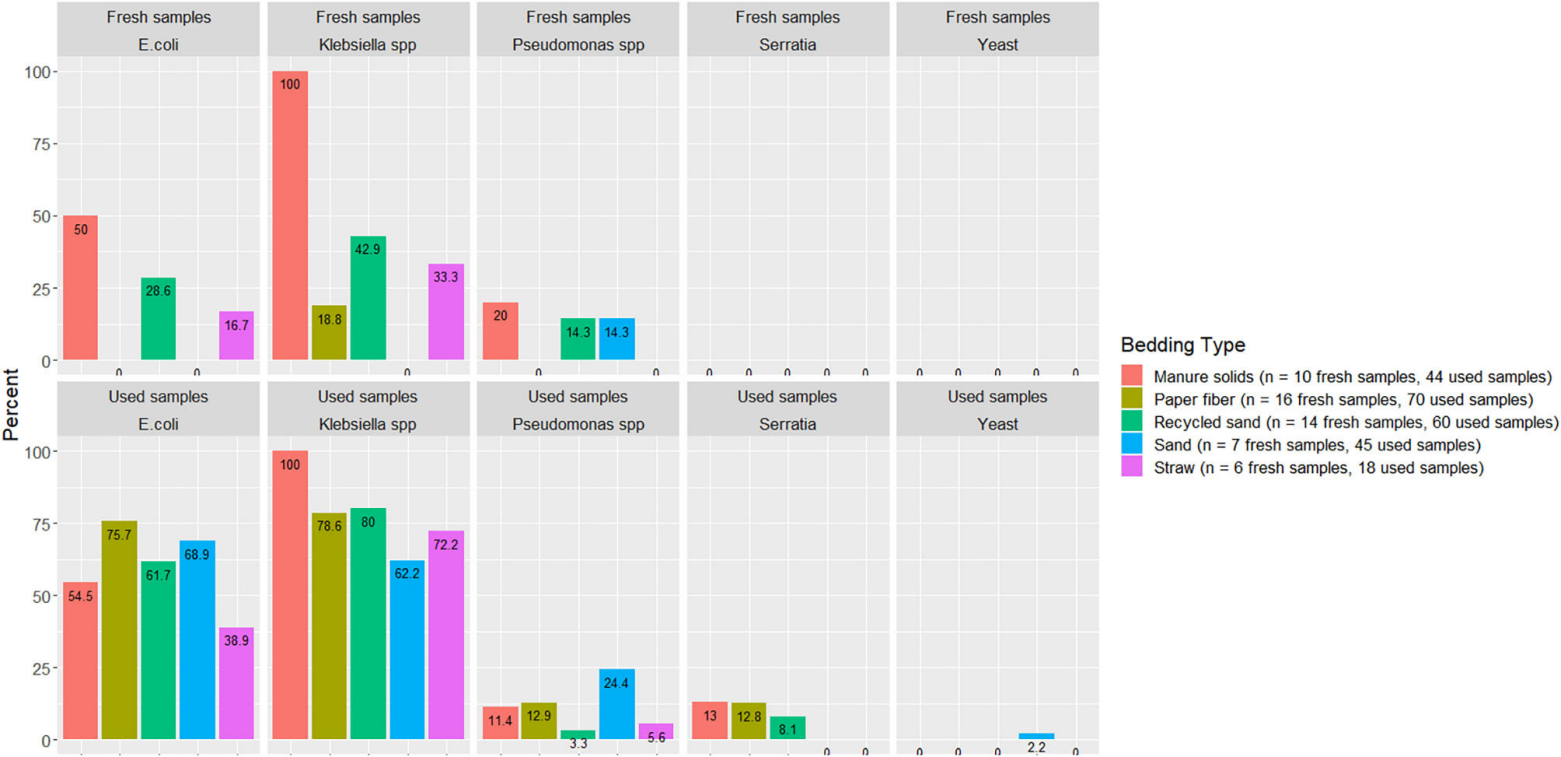
Proportion of used or fresh bedding samples with detectable organisms of mastitis importance. Fresh and used bedding samples collected from July 2018 to July 2019 from five conveniently selected New York dairy herds using one of five bedding materials in lactating pens. One fresh bedding sample and three to five used bedding samples were collected monthly following an SOP at each visit (unless there was no bedding available due to lack of supply or equipment malfunction on the follow-up visit).

A summary of the proportion of bedding samples in which bacteria were positively identified is shown in Figure 3 (i.e., if bacteria were not detected in fresh or used bedding these bacteria are not included in the figure).

### DM Content and pH

**Figure 4:**
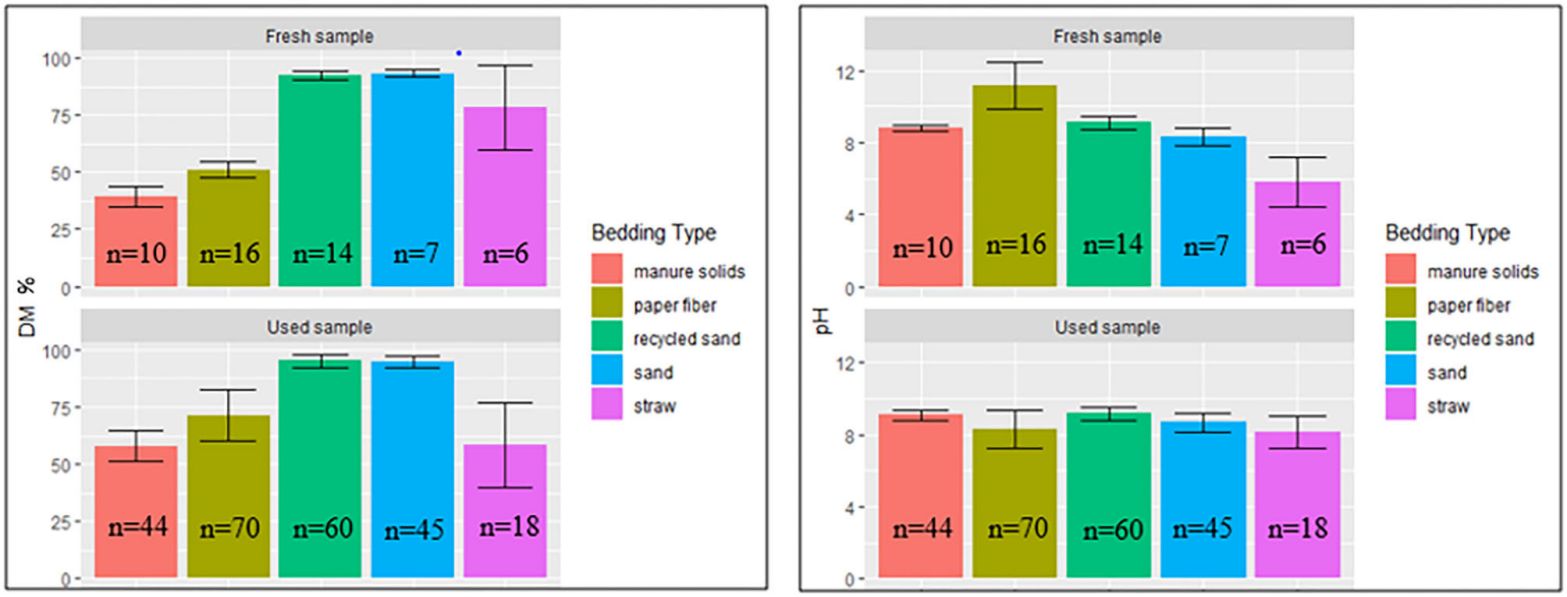
Average DM content (% DM) and pH values (Error bars represent SD) for fresh and used bedding samples collected from July 2018 to July 2019 from five conveniently selected New York dairy herds using one of five bedding materials in lactating pens. One fresh bedding sample and three to five used bedding samples were collected monthly following an SOP at each visit (unless there was no bedding available due to lack of supply or equipment malfunction on the follow-up visit).

**Figure 5:**
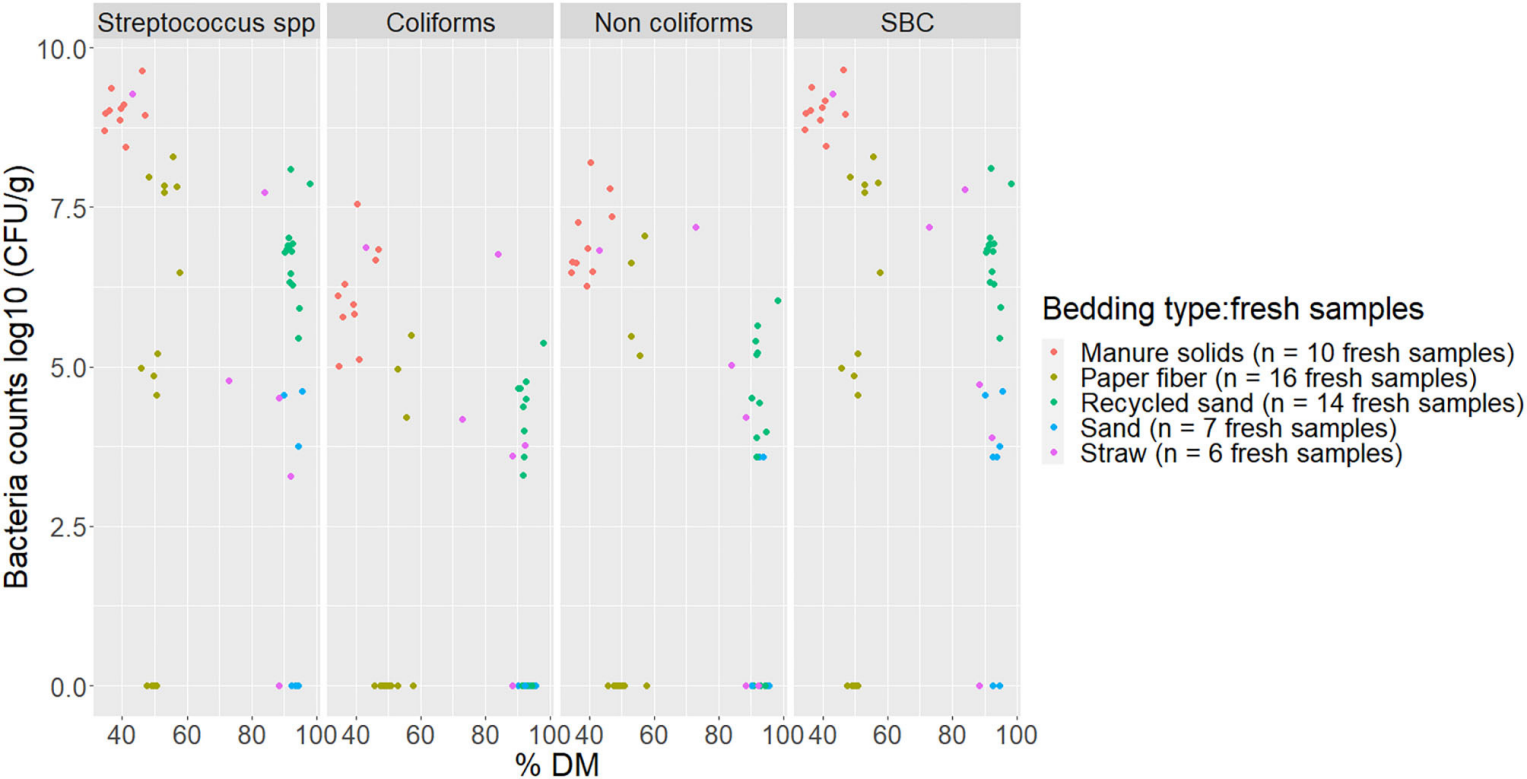
Scatterplot of DM content (% DM) vs. bacteria counts (log10 CFU/g) by bacteria group from fresh samples collected from July 2018 to July 2019 from five conveniently selected New York dairy herds using one of five bedding materials in lactating pens. One fresh bedding sample was collected monthly following an SOP at each visit (unless there was no bedding available due to lack of supply or equipment malfunction on the follow-up visit). When no bacteria were identified, a value of log_10_ + 1 CFU/g was given, assuming that at least 10 CFU were present. SBC (sum bacterial count) = *Streptococcus* spp, coliforms and non-coliforms summed.

**Figure 6:**
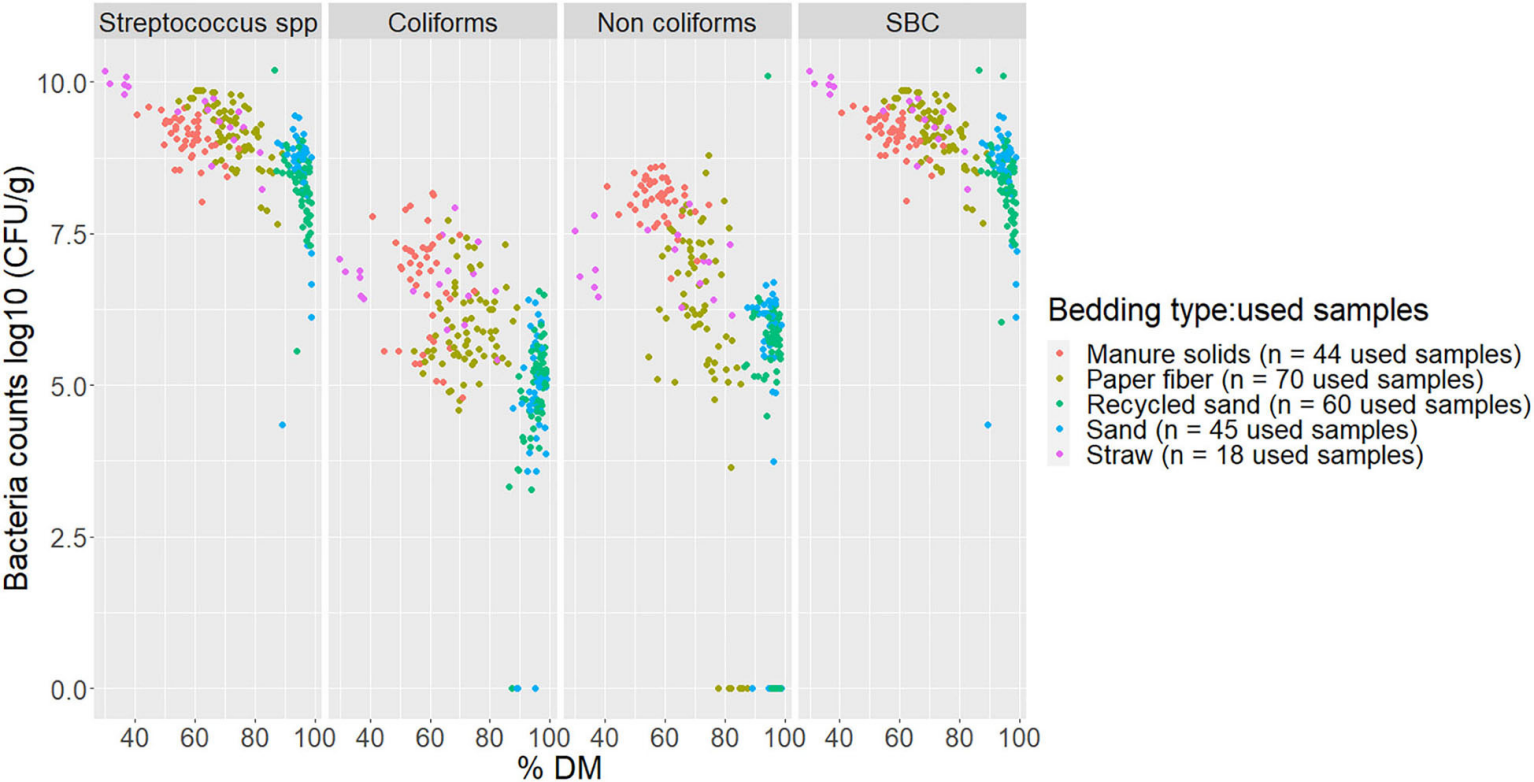
Scatterplot of DM content (% DM) vs. bacteria counts (log10 CFU/g) by bacteria group and bedding type in used bedding samples collected from July 2018 to July 2019 from five conveniently selected New York dairy herds using one of five bedding materials in lactating pens. Three to five used bedding samples were collected monthly following an SOP at each visit (unless there was no bedding available due to lack of supply or equipment malfunction on the follow-up visit). When no bacteria were identified, a value of log_10_ + 1 CFU/g was given, assuming that at least 10 CFU were present. SBC (sum bacterial count) = *Streptococcus* spp, coliforms and non-coliforms summed.

The percentage of DM content and pH values for fresh and used bedding samples during the entire study period are shown in Figure 4. Generally, inorganic bedding samples were dryer than organic. Regarding pH values, fresh samples were on the alkaline side within a range of 8–11 except for ST, with acidic values (5.8 ± 1.4). For used bedding samples, all materials were in the alkaline range of 8–9. Relationships between DM content and bacterial count in fresh and used samples are shown in Figures 5, 6, respectively. For example, correlation analysis showed a negative linear relationship between DM content and bacterial count in used samples: SBC (*r* = −0.61, *P* < 0.001), *Streptococcus* spp (*r* = −0.60, *P* < 0.001), coliforms (*r* = −0.56, *P* < 0.001) and for non-coliforms (*r* = −0.53, *P* < 0.001), suggesting drier bedding material had lower bacterial counts.

### BT Bacterial Counts

On several visits (*n* = 15), the BT had recently been picked up, and a BT sample was not available. A total of 40 BT samples were collected for the final analysis: MS (*n* = 11), PF (*n* = 7), RS (*n* = 8), SD (*n* = 8), and ST (*n* = 6).

**Figure 7:**
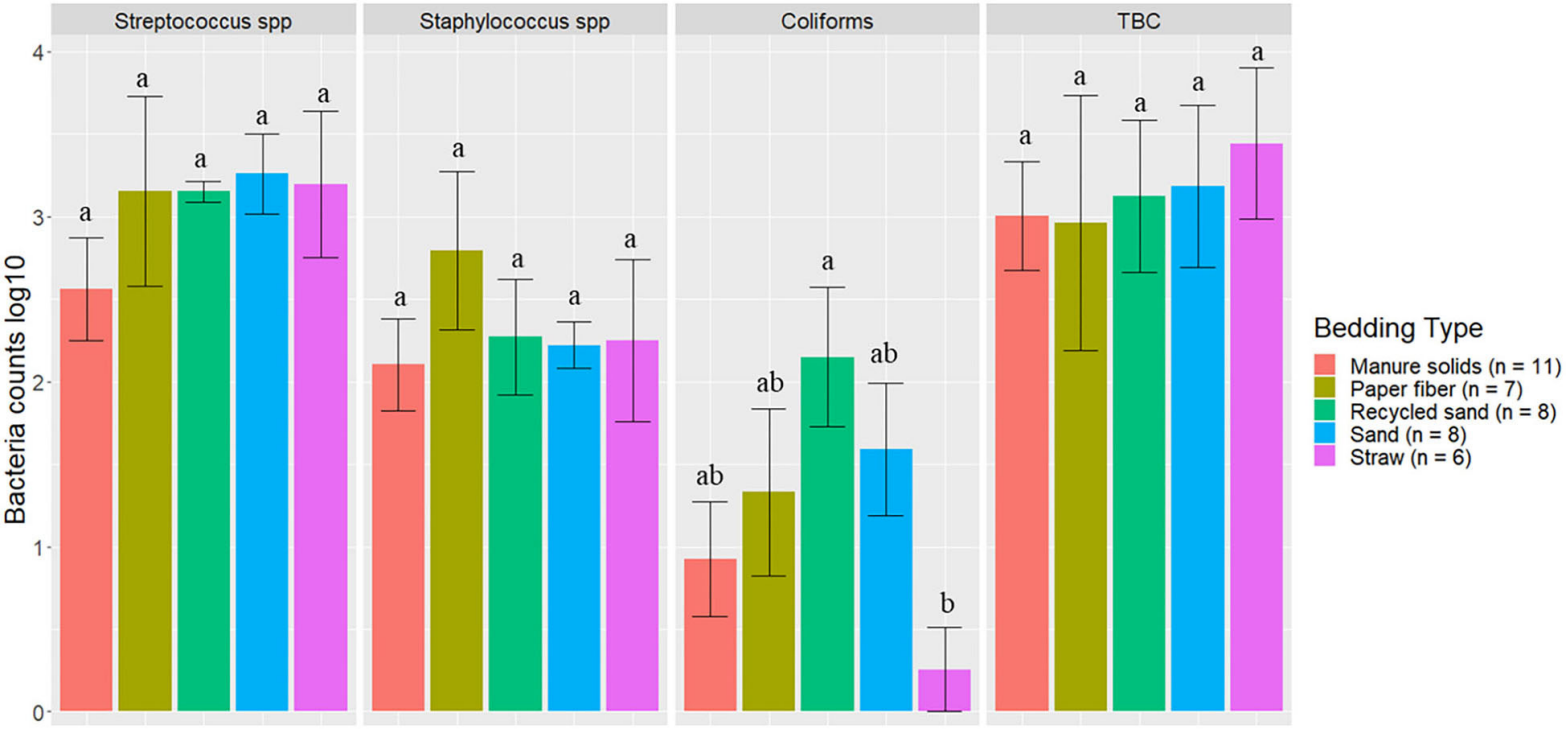
Average bacteria counts (log10 CFU/ml) in milk samples collected monthly (unless milk had been picked up prior to arrival for follow up visit) from the BT after mechanically agitating the milk for at least 5 min until sufficient homogeneity is obtained from five conveniently selected New York dairy herds using one of five bedding materials in lactating pens. Error bars represent SD. TBC = total bacteria count. When no bacteria were identified, a value of log_10_ + 1 CFU/ml was given, assuming that at least 10 CFU were present.

The bacterial groups evaluated in BT samples are summarized in Figure 7. Dunn's test with Bonferroni correction for multiple comparisons indicated that coliform counts on the ST herd (0.19) were observed to be different from those on RS (2.24) (*P* = 0.04) although it is important to notice that only one BT sample from this herd had detected levels of this bacterial group. In the other bacterial groups among herds based on the Kruskal–Wallis test, the *p*-values were *Streptococcus* spp (*P* = 0.19), *Staphylococcus* spp (*P* = 0.08), TBC (*P* = 0.57).

A correlation analysis of the average of coliforms and *Streptococcus* spp counts in used bedding samples and those counts in BT was performed, and the results showed a limited association with values of−0.09 (*P* = 0.5) and 0.06 (*P* = 0.6), respectively.

### Detection of Specific Bacteria in BT

**Figure 8:**
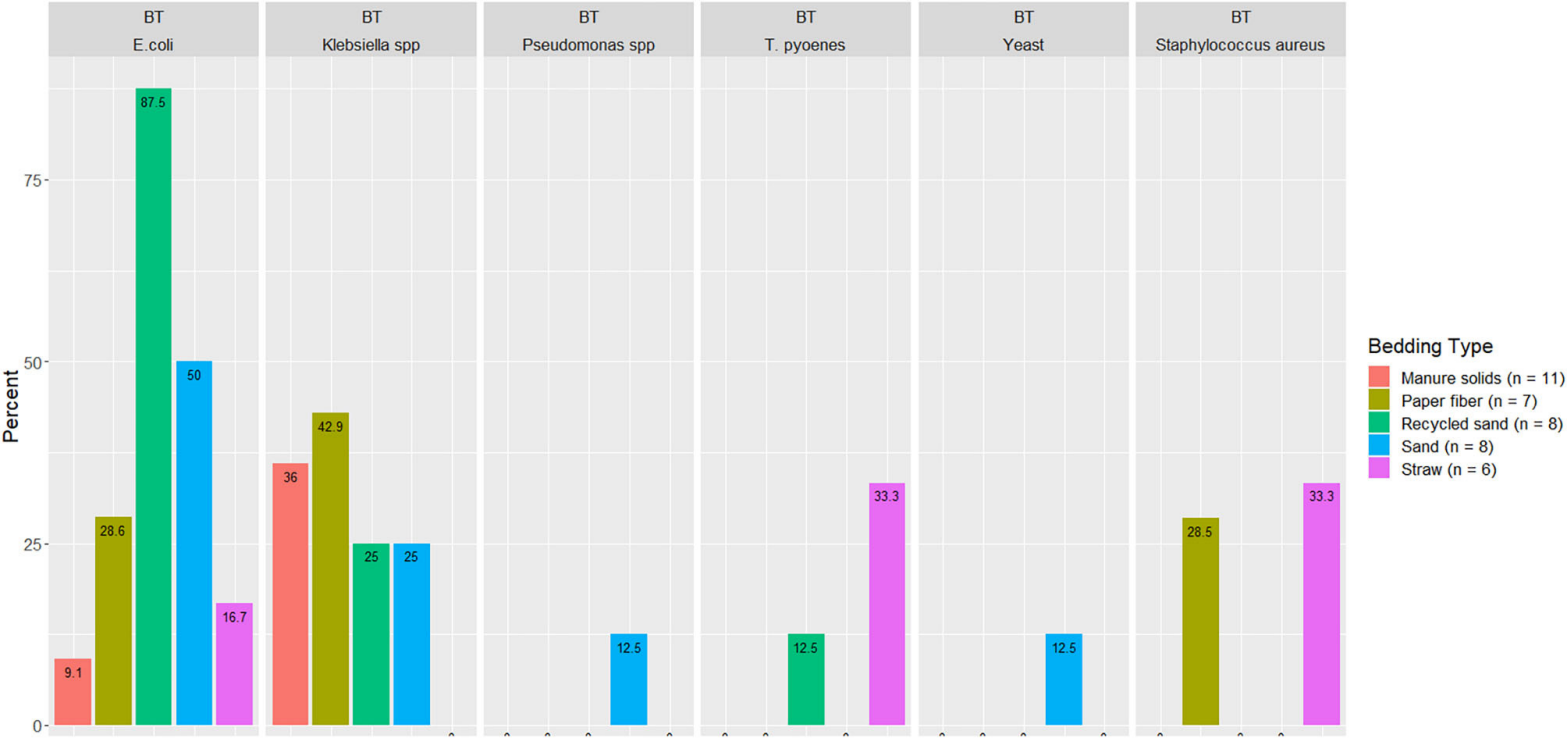
Displayed only the proportion from BT milk samples with detectable organisms of mastitis importance. Milk samples collected monthly (unless milk had been picked up prior to arrival for follow up visit) from the BT after mechanically agitating the milk for at least 5 min until sufficient homogeneity is obtained from five conveniently selected New York dairy herds using one of five bedding materials in lactating pens.

A summary of the proportion of BT samples with detectable pathogens of mastitis importance are illustrated in Figure 8 (i.e., those without detectable organisms are not displayed).

### BT Somatic Cell Linear Score

**Figure 9:**
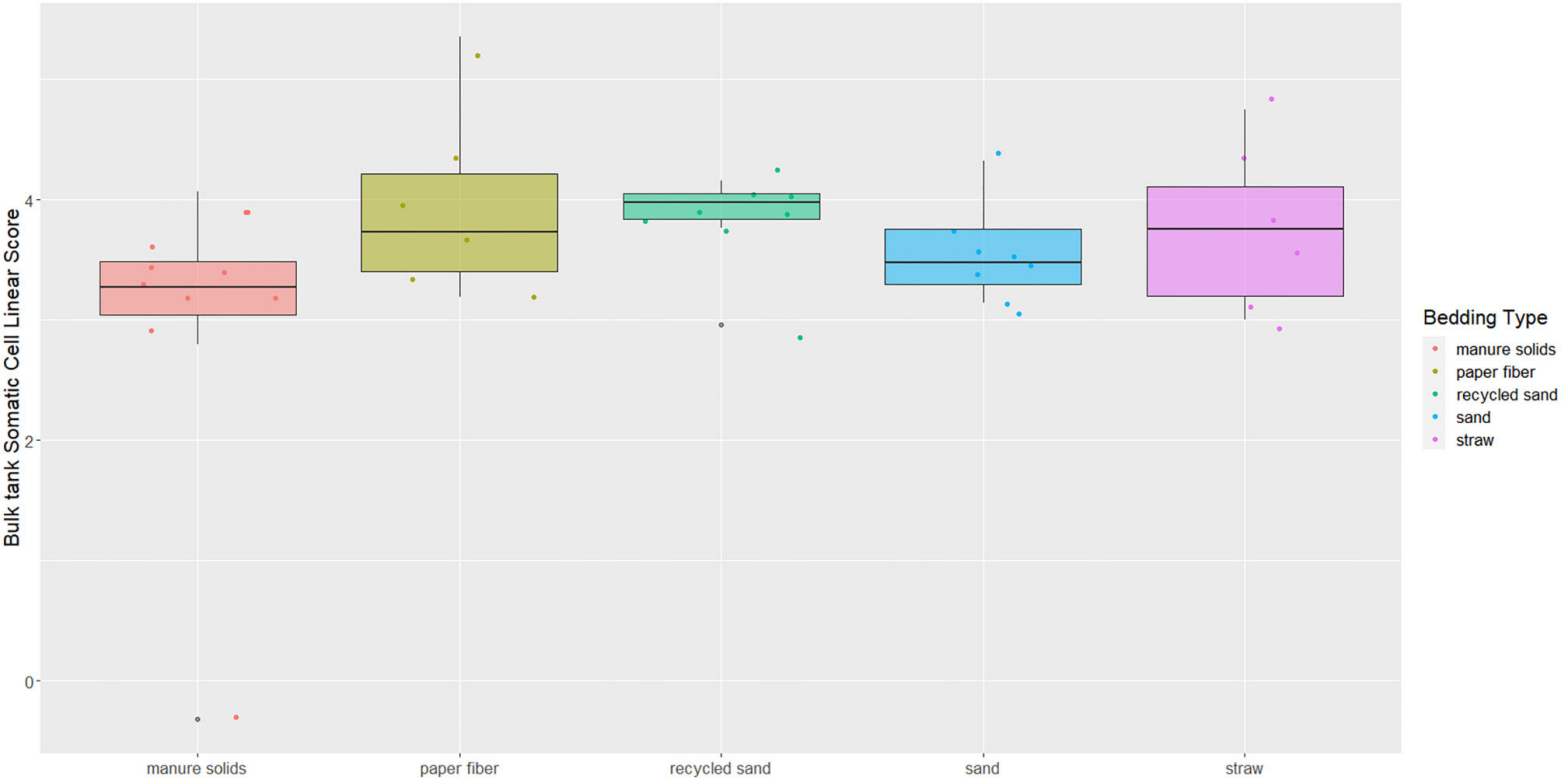
Boxplots showing 25th, 50th (median), and 75th percentiles of somatic cell score in milk samples monthly collected (unless milk had been picked up prior to arrival for follow up visit) from the BT after mechanically agitating the milk for at least 5 min until sufficient homogeneity is obtained from five conveniently selected New York dairy herds using one of five bedding materials in lactating pens. Milk samples analyzed using DCC to get SCCs and transformed into somatic cell scores (BTSLS) by applying the following equation: BTSLS = log2 (BTSCC/100) + 3.

The overall BTSLS among herds was 3.54, ranging from 2.80 to 5.35 (Figure 9). The *p*-value for the Kruskal–Wallis test for the differences observed among bedding materials and BTSLS was 0.13.

## Discussion

This study describes characteristics (i.e., bacteria counts, pH, and DM) for fresh and used bedding samples as well as bacterial counts and SCCs from BT samples. These samples were collected, following a strict sampling SOP, monthly over 1 year from five conveniently selected New York dairy farms. Each farm used one of five bedding materials in lactating pens. In addition to describing these characteristics, this sampling scheme allowed us to demonstrate the variability within bedding samples in the same farm.

The first objective of this study was to describe bedding bacterial counts, pH, and DM. It is known that bedding material (especially organic material) can support bacterial growth due to contained nutrients, and even inorganic bedding once soiled with feces, urine, or any other cow secretion can grow bacteria. Our results confirm this with bacterial counts higher in used samples compared with fresh samples, which agrees with what has been stated by other research groups (29, 30, 32, 33). Evaluating these bedding characteristics is important because organic bedding material has been associated with higher bacterial load (30, 32) and with higher bacterial counts on teat skin (22, 23). Our results on bacterial counts were generally highest for MS on fresh bedding samples for all bacterial groups, which is similar to what is described by other researchers (23, 30). Particularly, coliforms counts were not different between RS, ST, and PF. As for used samples, we observed that organic materials supported the highest levels of all bacterial groups (Figure 1). In herds bedding with inorganic bedding material, *Streptococcus* spp levels were lowest in RS compared with SD, but similar to previous research (34), there was no difference in the number of coliforms and non-coliforms.

Several organisms of mastitis importance were not quantified but rather their presence evaluated because we focused on the pathogens that we can manage in bedding. In other words, we manage all *Streptococcus* species as a whole, but we cannot specifically manage *Streptococcus uberis* or *Streptococcus dysgalactiae*. *E. coli* was detected in only 50% and 54% of MS fresh and used bedding samples, respectively, which was surprising given that *E. coli* is known to exist in high quantities in feces. Apparently, in the herd studied here that bedded with MS, the manure and bedding processing procedures reduced *E. coli* to levels below detection. However, another fecally shed organism, *Klebsiella* spp, was found in 100% of fresh and used MS samples (Figure 3), suggesting that, at least on this farm, the manure processing and bedding procedures did not eliminate *Klebsiella* spp, leaving it as a risk factor for intramammary infections.

In our study, DM content was higher for RS and SD (~92%) in fresh samples, similar to Canadian farms (35) and within the ranges reported for used samples (~95%) by Zdanowicz, Patel, and Kristula (22, 30, 34). These values seem to have low variability across studies. Fresh MS had a DM average content of 39.5% (34.2–47.0%), similar to the values reported by Robles (35). However, a different study (30) reports a much wider range (21.4 – 96.3%) in samples collected from 17 states across the United States. That variability might be explained by different MS processing techniques (i.e., digested, compost, or fresh) and possibly due to the sampling variability (e.g., time in relation to when were applied). In our study, used MS samples had a higher DM content (57.8%, range of 40.6–74.6%) compared to MS fresh samples. This observation has been reported by others (35–37).

The relationship of DM content and bacterial growth suggest that drier bedding material impedes bacterial growth for all bacterial groups in all bedding types. The correlations between these variables are similar to the ones reported by Zdanowicz (22). As a result, a high percentage of DM (e.g., as for RS or SD) supported the lowest levels of growth of *Streptococcus* spp, coliforms, and non-coliforms compared with those bedding materials with a lower percentage of DM (Figures 5, 6).

Regarding pH values, most of the bedding material samples were on the alkaline side within a range of 8–11 except for ST with acidic values (5.8 ±1.4) in fresh samples (Figure 4). This is similar to those reported in other research (30). This can be of importance when controlling some bacteria species that do not multiply well in low-pH environments (19).

Our results show that, even following a standardized sampling protocol, the bacterial count distribution in used samples within the same day of sampling had a noticeable variation, especially in PF, RS, and ST materials. The MS and SD appeared to have counts that were more constant within the same day of sampling although they differed throughout the study period (Figure 2). This suggests that using summarized data, such as averages might not be a good way to analyze bedding bacteria because one might lose a lot of important information about the variability. This is important to consider when evaluating bedding samples and a specific outcome and when using only a few samples from a specific point in time in an attempt to describe bedding data.

The second objective was to evaluate the association between bedding type with milk quality. When evaluating the association between BT bacteria and bedding bacteria counts, our results show the greatest difference was in coliforms in the RS and ST bedding (Figure 7). However, ST is also the bedding from the farm that was only present for 6 months of the study, so these findings should be interpreted with caution. Other studies show a similar lack or marginal association between BT total bacterial count and bedding type (27, 28), respectively. Bradley reported a marginal difference, and it was higher for farms using recycled MS bedding, followed by wood products, ST, and SD. The detected organisms of mastitis importance varied across BT samples; surprisingly, *E. coli* was detected in only 9.1% of samples from the MS herd, whereas it was 87.5% in RS farm (Figure 8). We did not find an association between bedding type and BTSLS (Figure 9).

It is important to note that other cross-sectional bedding studies used only two points in time during different seasons (winter and summer) and did not take into account the variability during an extended period. Even though these researchers showed the variability among farms, they did not take into account the variability in bacterial counts within the same farm, on the same day of sampling, or even how the sampling method can affect these parameters.

## Strengths and Limitations

This was a descriptive study that prospectively evaluated bedding bacterial counts over time. The two main strengths of this study are the consistent sampling SOP and serial sampling of bedding through time. These features can reduce the variability in sample procurement and improve the understanding of bedding bacteria count variability among sampling times.

However, missing bedding and BT samples decreased the number of complete evaluations. Another possible limitation is the use of frozen samples, which can result in possible measurement error in bacterial counts. Although Homerosky (38) reports a decrease in Gram-negative and coliform bacteria counts after freezing, the QMPS laboratory did not find any difference in bacteria counts. In the aforementioned data from QMPS, bacteria counts were evaluated weekly from 20 bedding samples and did not show a significant difference between each day for up to 21 days (M. Zurakowski, unpublished data).

Finally, this study only involved five herds, each with one bedding type. Thus, only one experimental unit per bedding type was included in this analysis, and this limits the ability to generalize the findings to other farms using these types of bedding material. Nonetheless, our results show that even conducting repeated sampling within a farm, there was a significant variation in the bacterial count within the sampling day and throughout the study period (monthly samples). These findings indicate that results from studies evaluating the association between bedding material and bulk tank bacterial load should be interpreted with caution, especially if a single or few sample collections were carried out over time. That may be a concern even in studies enrolling several herds per bedding material.

The herds enrolled in our study were well-managed and conveniently selected; therefore, our findings should not be generalized to herds with different management practices and different bedding processing. Differences in management practices in each herd may likely influence the bedding bacterial counts and the association between bedding type and BT parameters. However, it is important to mention that the main objective of this study was to report the variability in bacterial counts within the farms over time and its association with the bacterial load present in the BT milk. The association assessment between bedding bacterial counts and particular herd management practices was not in the scope of the study.

## Conclusions

Bedding sample results can be difficult to interpret because bacteria counts in bedding are not easily linked to bacteria counts in BT or milk quality. Results from this study show that there is a lot of variability in bedding samples even when collected under strict SOP guidelines. In bedding samples, a higher DM content had the lowest levels of bacterial growth compared with those with lower DM content. No associations between BT bacteria counts and bedding bacterial counts were observed. No association between BTSCC based on bedding type were observed. Despite using an SOP for bedding sampling in an effort to consistently collect samples, we still observed a large amount of variability both within and among bedding samples. This variability may have obscured any potential association between BT milk quality and bedding type.

## Data Availability Statement

The raw data supporting the conclusions of this article will be made available by the authors, without undue reservation.

## Author Contributions

VA and PO conceived the presented idea. PO directed the project. VA performed all sampling. MZ and DP performed the laboratory analysis. VA analyzed the data. TT, DN, and PO encouraged VA to investigate different ways to analyze the data. VA interpreted the results and wrote the manuscript. All authors contributed to the article and approved the submitted version.

## Conflict of Interest

PO was employed by the company Lechear LLC. The remaining authors declare that the research was conducted in the absence of any commercial or financial relationships that could be construed as a potential conflict of interest.
